# Supplementary material for: BNT162b2 vaccine induces antibody release in saliva: a possible role for mucosal viral protection?
Source: EMBO Mol Med. 2022 Apr 19;14(5):e15326. doi: 10.15252/emmm.202115326 (PMC9081904; doi:10.15252/emmm.202115326)
Supplement: Supplementary file 1 — Appendix [file EMMM-14-e15326-s005.pdf]

Table of Content

Appendix Table S1. F1-scores computed for different threshold levels.

Appendix Table S2. Demographic table.

Appendix Figure S1. SARS-CoV-2 specific antibody response in plasma up to three months after BNT162b2 vaccination.

Appendix Figure S2. SARS-CoV-2 specific antibody response in saliva up to three months after BNT162b2 vaccination.

Appendix Table S1. F1-scores computed for different threshold levels.

Treshold	Sensitivity%	Specificity%	F1-Score
0,0485	100,00	1,35	2,67
0,0535	100,00	2,70	5,26
0,058	100,00	4,05	7,79
0,0635	100,00	5,41	10,26
0,071	100,00	6,76	12,66
0,0835	100,00	8,11	15,00
0,098	100,00	9,46	17,28
0,106	100,00	10,81	19,51
0,11	100,00	12,16	21,68
0,116	100,00	13,51	23,80
0,1205	100,00	14,86	25,87
0,123	100,00	16,22	27,91
0,126	100,00	17,57	29,89
0,135	100,00	20,27	33,71
0,1445	100,00	24,32	39,12
0,1505	100,00	25,68	40,87
0,1615	100,00	27,03	42,56
0,1745	100,00	28,38	44,21
0,209	100,00	29,73	45,83
0,2395	100,00	31,08	47,42
0,242	100,00	32,43	48,98
0,246	100,00	33,78	50,50
0,2605	100,00	35,14	52,01
0,2815	100,00	36,49	53,47
0,292	100,00	37,84	54,90
0,298	100,00	39,19	56,31
0,3105	100,00	40,54	57,69
0,332	100,00	41,89	59,05
0,3555	100,00	43,24	60,37
0,3695	100,00	44,59	61,68
0,3775	100,00	45,95	62,97
0,3875	100,00	47,30	64,22
0,3955	100,00	50,00	66,67
0,412	100,00	51,35	67,86
0,445	100,00	52,70	69,02
0,4655	100,00	54,05	70,17
0,4695	100,00	55,41	71,31
0,494	100,00	56,76	72,42
0,5255	100,00	58,11	73,51
0,58	100,00	59,46	74,58
0,6465	100,00	60,81	75,63
0,6705	100,00	62,16	76,67
0,6905	100,00	63,51	77,68
0,721	100,00	64,86	78,68
0,769	100,00	66,22	79,68
0,8205	100,00	67,57	80,65
0,8535	100,00	68,92	81,60
0,884	100,00	70,27	82,54
0,914	100,00	71,62	83,46
0,9435	100,00	72,97	84,37
1,024	100,00	74,32	85,27
1,161	100,00	75,68	86,16
1,237	100,00	77,03	87,02
1,344	100,00	78,38	87,88
1,512	100,00	79,73	88,72
1,595	100,00	81,08	89,55
1,627	100,00	82,43	90,37
1,687	100,00	83,78	91,17
1,784	100,00	85,14	91,97
1,893	100,00	86,49	92,76
2,016	98,65	86,49	92,17
2,097	97,30	86,49	91,58
2,187	97,30	87,84	92,33
2,261	95,95	87,84	91,72

Treshold	Sensitivity%	Specificity%	F1-Score
2,293	94,59	87,84	91,09
2,325	93,24	87,84	90,46
2,348	91,89	87,84	89,82
2,378	91,89	89,19	90,52
2,386	90,54	89,19	89,86
2,396	90,54	90,54	90,54
2,431	90,54	91,89	91,21
2,46	87,84	91,89	89,82
2,473	87,84	93,24	90,46
2,486	86,49	93,24	89,74
2,504	85,14	93,24	89,01
2,521	83,78	93,24	88,26
2,549	82,43	93,24	87,50
2,578	81,08	93,24	86,74
2,601	79,73	93,24	85,96
2,629	78,38	93,24	85,17
2,654	77,03	93,24	84,36
2,688	75,68	93,24	83,55
2,728	74,32	93,24	82,71
2,775	72,97	93,24	81,87
2,804	71,62	93,24	81,01
2,812	70,27	93,24	80,14
2,826	68,92	93,24	79,26
2,849	67,57	93,24	78,36
2,886	67,57	94,59	78,83
2,926	66,22	94,59	77,90
2,948	64,86	94,59	76,95
2,956	63,51	94,59	76,00
2,97	62,16	94,59	75,02
2,985	60,81	94,59	74,03
2,999	59,46	94,59	73,02
3,015	58,11	94,59	71,99
3,021	56,76	94,59	70,95
3,042	55,41	94,59	69,88
3,074	54,05	94,59	68,79
3,094	52,70	94,59	67,69
3,113	51,35	94,59	66,56
3,131	50,00	94,59	65,42
3,147	48,65	94,59	64,25
3,159	47,30	94,59	63,06
3,162	45,95	94,59	61,85
3,168	44,59	94,59	60,61
3,176	43,24	94,59	59,35
3,185	41,89	94,59	58,07
3,193	40,54	94,59	56,76
3,198	39,19	94,59	55,42
3,211	37,84	94,59	54,06
3,23	36,49	94,59	52,66
3,246	35,14	94,59	51,24
3,262	33,78	94,59	49,78
3,283	32,43	94,59	48,30
3,303	31,08	94,59	46,79
3,347	29,73	94,59	45,24
3,389	28,38	94,59	43,66
3,421	27,03	94,59	42,05
3,464	25,68	94,59	40,39
3,486	24,32	94,59	38,69
3,493	22,97	94,59	36,96
3,511	21,62	94,59	35,20
3,53	20,27	94,59	33,39
3,539	18,92	94,59	31,53
3,549	17,57	94,59	29,64
3,554	16,22	94,59	27,69
3,558	14,86	94,59	25,68
3,573	13,51	94,59	23,64
3,607	12,16	94,59	21,55

Treshold	Sensitivity%	Specificity%	F1-Score
3,637	10,81	94,59	19,40
3,658	10,81	95,95	19,43
3,683	9,46	95,95	17,22
3,722	8,11	95,95	14,95
3,775	6,76	95,95	12,62
3,812	5,41	95,95	10,23
3,843	4,05	95,95	7,78
3,923	2,70	95,95	5,26
4,028	1,35	95,95	2,66
4,233	0,00	95,95	0,00
5,161	0,00	97,30	0,00
6,665	0,00	98,65	0,00

Appendix Table S2. Demographic table.

	Cohort	Counts	Mean	Std. Dev	Std. Error of Mean
Serology from symptom onset (Days)	COVID-19	28	23,9	9,6	1,8
Age	COVID-19	28	47,3	16,6	3,1
	Control	19	42,8	15,4	3,5
	Vaccinated (Plasma)	92 (21 SARS-CoV-2 Exp)	38,2	11,9	1,2
	Vaccinated (Saliva)	85 (18 SARS-CoV-2 Exp)	38,5	12	1,3
Male	COVID-19	9			
	Control	7			
	Vaccinated (Plasma)	34			
	Vaccinated (Saliva)	36			
Female	COVID-19	19			
	Control	12			
	Vaccinated (Plasma)	58			
	Vaccinated (Saliva)	49			

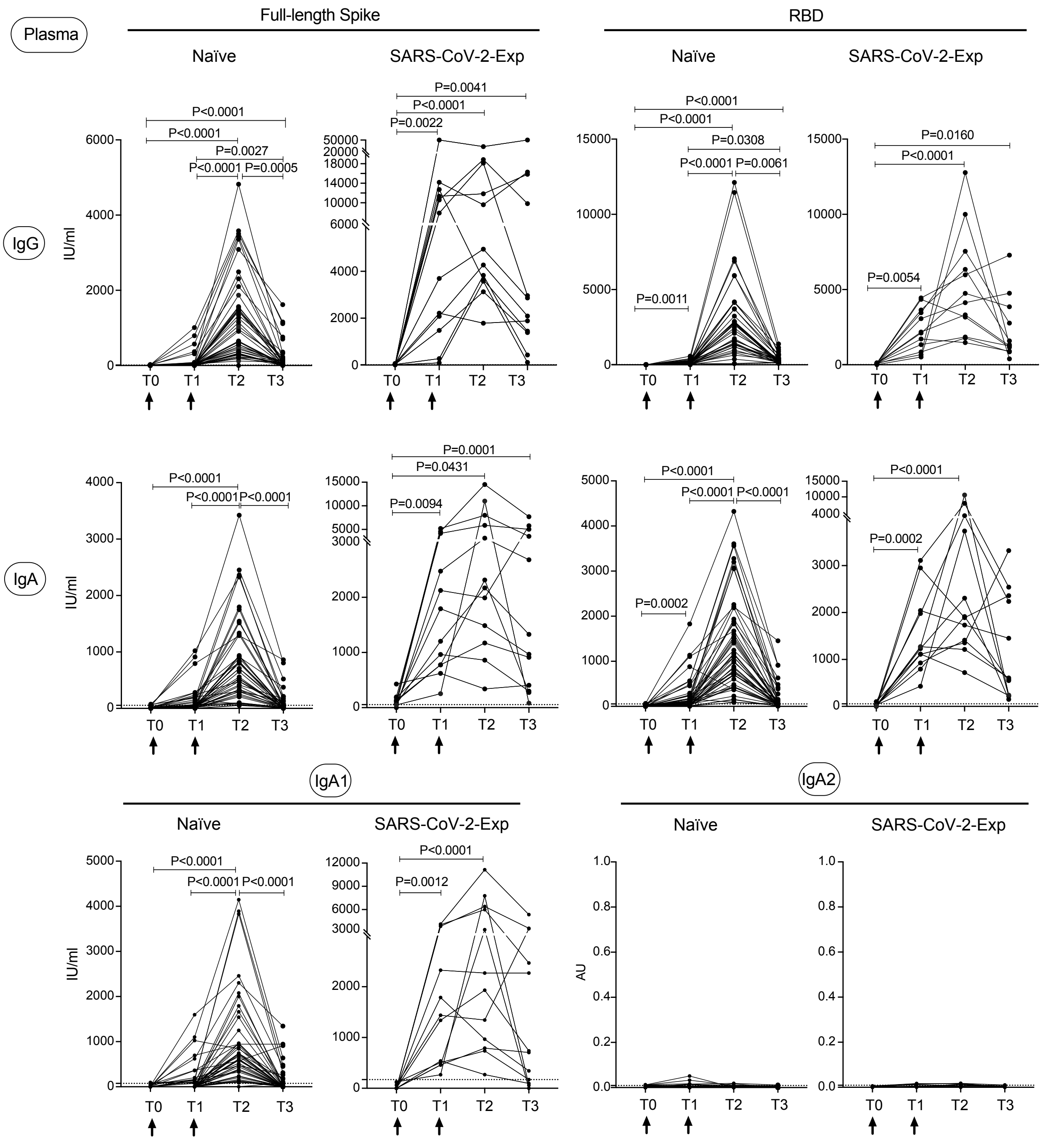

Appendix Figure S1. SARS-CoV-2 specific antibody response in plasma up to three months after BNT162b2 vaccination. SARS-CoV-2 specific antibody responses were measured in the plasma of vaccinated naïve (n=44) and SARS-CoV-2-Exp (n=12) subjects at different time points: at the time of the first (T0) and the 2nd (T1) vaccine dose (indicated with an arrow), at 7-10 days (T2) and 3 months (T3) after the 2nd dose. Plasma was tested for IgG and IgA to full-length spike and its receptor binding domain (RBD) and for anti-spike IgA1 and IgA2. The titers of antigen specific Ig are expressed in IU/ml, except for IgA2, expressed in AU (see Methods). LoD is indicated by a dotted line: LoD (spike IgG)=12, LoD (RBD IgG)=13.8, LoD (spike IgA)=54.22, LoD (RBD IgA)=54.08, LoD (IgA1)=74.64; LoD (IgA2)=0.009. Spaghetti plots showing the trends for each individual subject by linked dots. Each dot corresponds to an individual subject. *P* values were determined using Friedman test with Dunnett's multiple comparisons test.

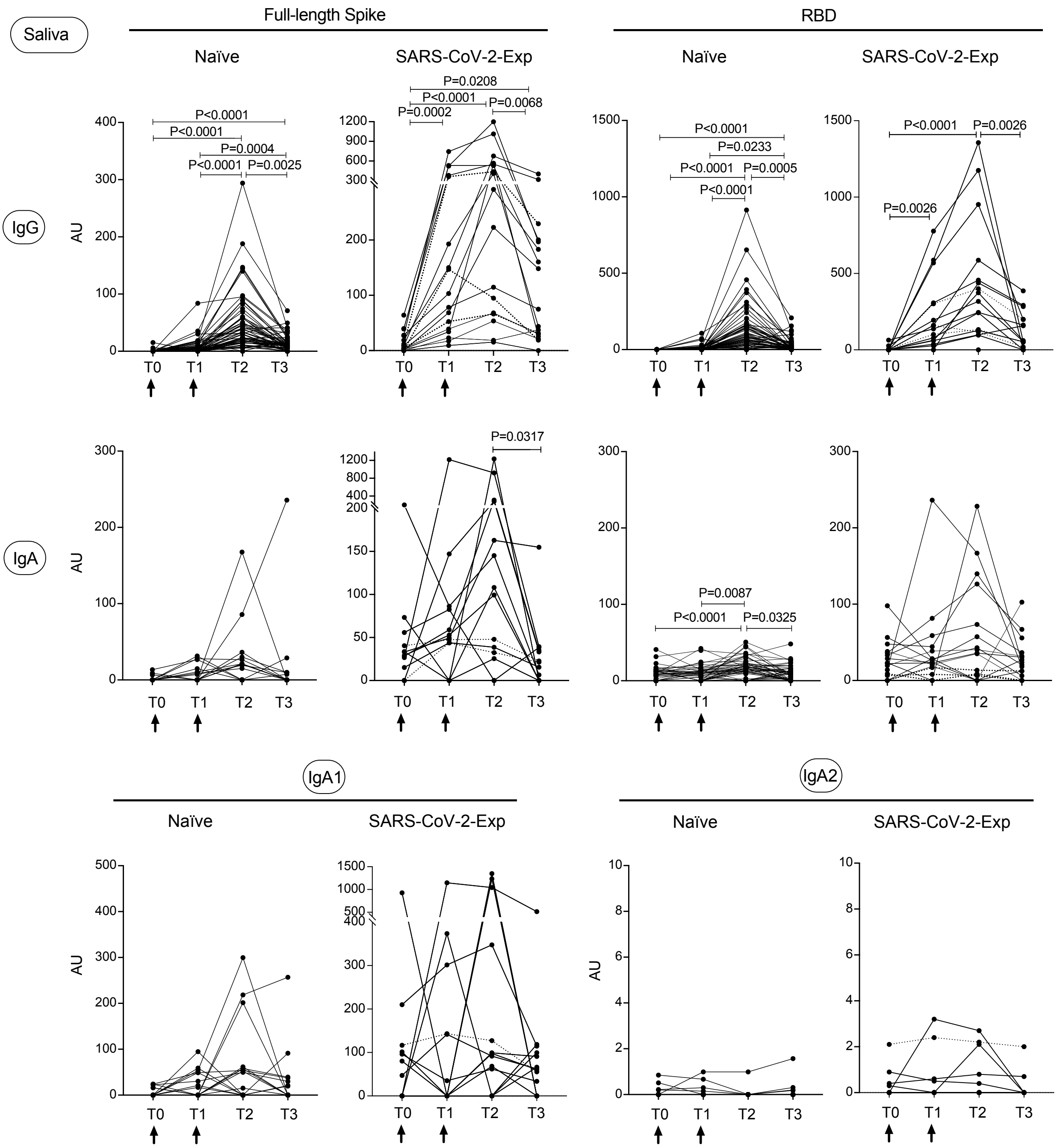

Appendix Figure S2. SARS-CoV-2 specific antibody response in saliva up to three months after BNT162b2 vaccination. SARS-CoV-2 specific antibody responses were measured in the saliva of vaccinated naïve (n=59) and SARS-CoV-2-Exp (n=17) subjects at different time points: at the time of the first (T0) and the 2nd (T1) vaccine dose (indicated with an arrow), at 7-10 days (T2) and 3 months (T3) after the 2nd dose. Saliva was tested for IgG and IgA to full-length spike and its receptor binding domain (RBD) and for anti-spike IgA1 and IgA2. The titers of antigen specific Ig were normalized by dividing the values of SARS-CoV-2 specific Ig by total IgA or total IgG concentrations of each sample. The normalization was applied only to values higher than LoD. The adjusted values are expressed in AU. Spaghetti plots showing the trends for each individual subject by linked dots. Each dot corresponds to an individual subject. Following the guidelines of the Italian Ministry of Health, some SARS-CoV-2-Exp subjects received only one dose of vaccine (dotted line). *P* values were determined using Friedman test with Dunnett's multiple comparisons test.
