# Supplementary material for: BNT162b2 vaccine induces antibody release in saliva: a possible role for mucosal viral protection?
Source: EMBO Mol Med. 2022 Apr 19;14(5):e15326. doi: 10.15252/emmm.202115326 (PMC9081904; doi:10.15252/emmm.202115326)
Supplement: Supplementary file 2 — Expanded View Figures PDF [file EMMM-14-e15326-s003.pdf]

Expanded View Figures

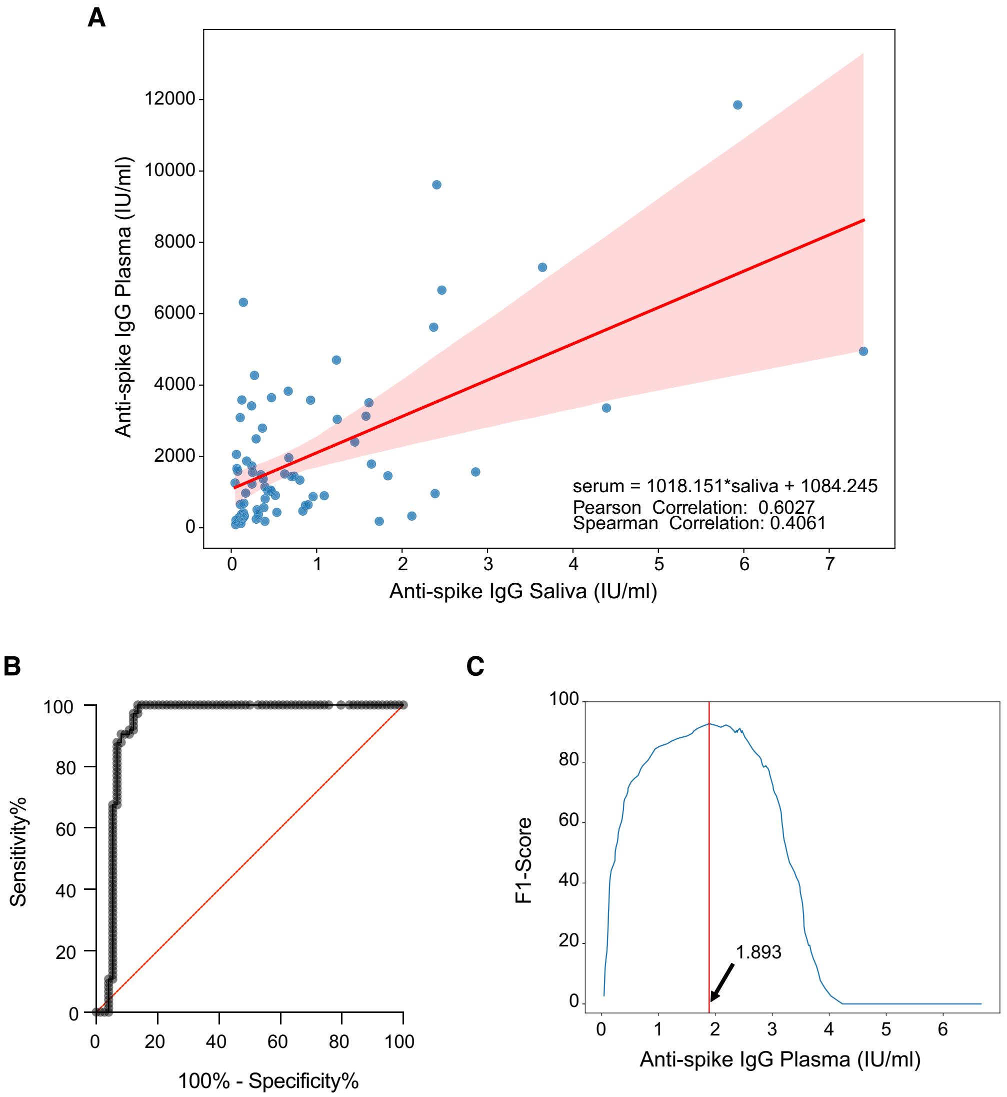

Figure EV1. Identification of saliva SARS-CoV-2-specific IgG threshold in relation to plasma SARS-CoV-2-specific IgG.

A Scatterplot of the recorded values of spike SARS-CoV-2-specific IgG in plasma and saliva, on the X-axis and Y-axis, respectively. In red, linear interpolation obtained by regression of SARS-CoV-2-specific IgG in the plasma on IgG in the saliva.

B ROC curve (0.9374 $\pm$ 0.02597, 95% CI 0.8865 to 0.9883 $P < 0.0001$).

C Plot of the F1 scores computed for different threshold levels. Log values of anti-spike IgG in the plasma are reported on X-axis. The vertical red line highlights the maximum F1 score registered and the respective threshold value: 1.893 (log plasma) that corresponds to 78,16 IU/ml.

© 2022 The Authors

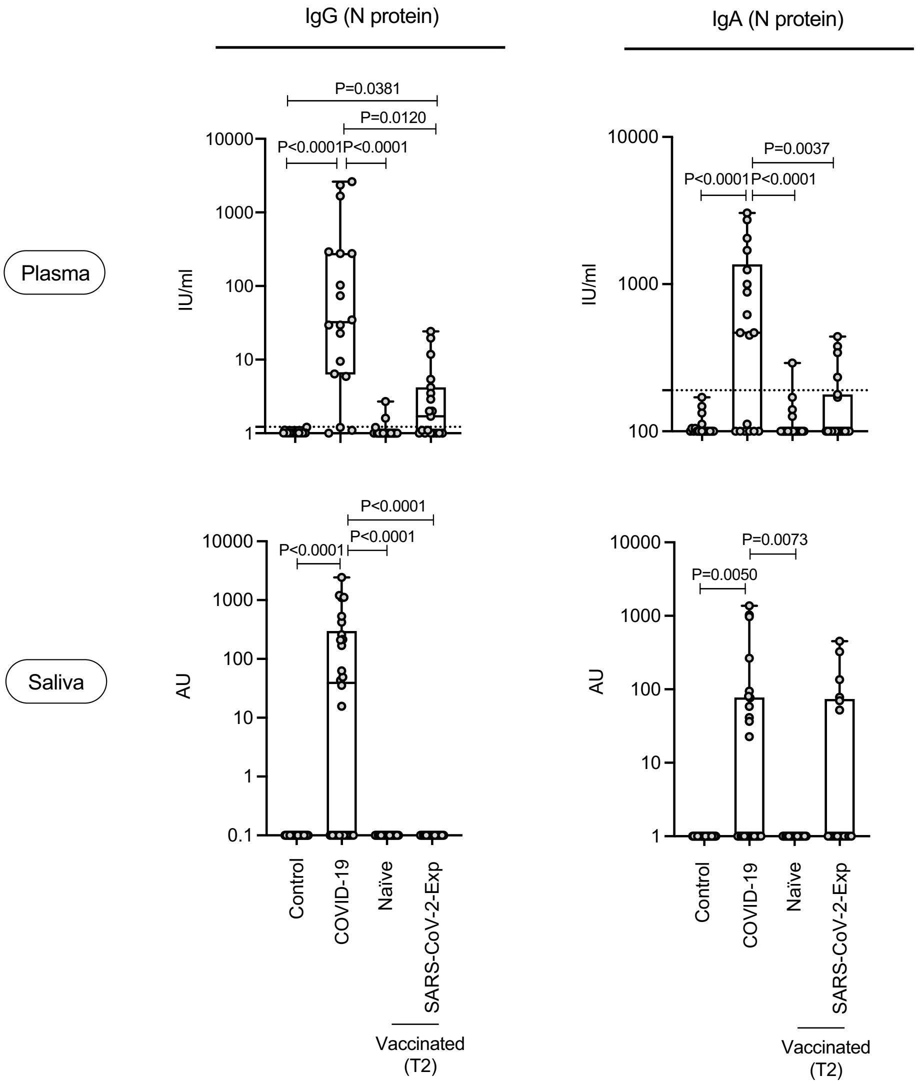

Figure EV2. IgG and IgA to nucleocapsid (N protein) in saliva and plasma.

Anti-N (nucleocapsid protein) IgG and IgA were measured both in plasma and saliva of vaccinated naïve (n = 18) and SARS-CoV-2-Exp (n = 19) subjects at 7–10 days after the second dose (T2), in COVID-19 patients (plasma: n = 19; saliva: n = 26) and in nonvaccinated subjects (n = 19, Control). For plasma samples, the titers of antigen-specific Ig are expressed in IU/ml. LoD is indicated by a dotted line: LoD (N IgG) = 1.22, LoD (N IgA) = 190. For saliva samples, the titers of antigen-specific Ig were normalized by dividing the values of SARS-CoV-2-specific Ig by total IgA or total IgG concentrations of each sample. The normalization was applied only to values higher than LoD. The adjusted values are expressed in AU. The box plots show the interquartile range, the horizontal lines show the median values, and the whiskers indicate the minimum-to-maximum range. Each dot corresponds to an individual subject. Log scale on y axis. P-values were determined using the Kruskal–Wallis test with the Dunn's multiple comparison test.

© 2022 The Authors

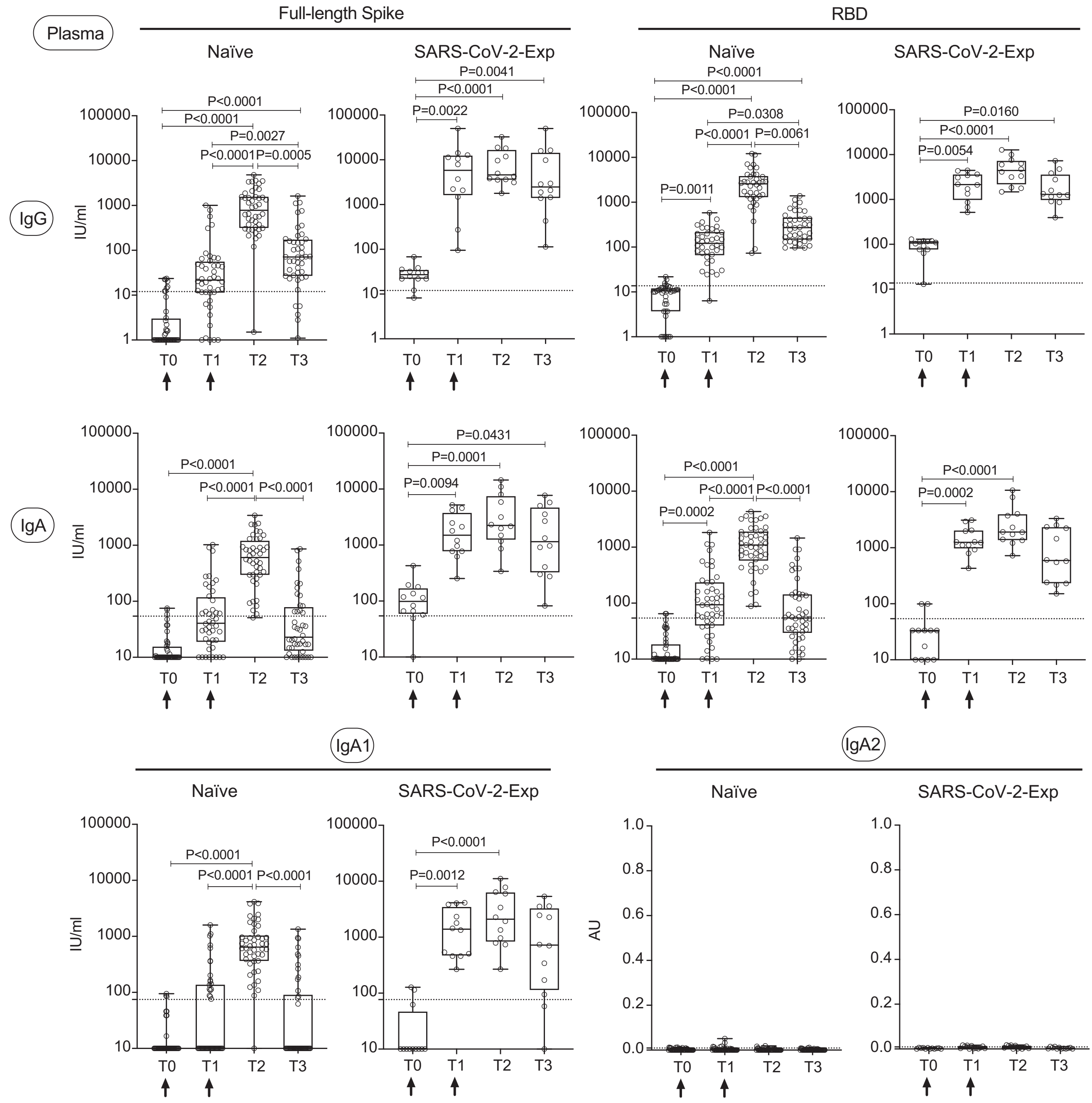

Figure EV3. SARS-CoV-2-specific IgG, IgA, and IgA1 levels decrease in plasma three months after BNT162b2 vaccination.

SARS-CoV-2-specific antibody responses were measured in the plasma of vaccinated naïve (n = 44) and SARS-CoV-2-Exp (n = 12) subjects at different time points: at the time of the first (T0) and the 2nd dose vaccine dose (T1) (indicated with an arrow), at 7–10 days (T2) and 3 months (T3) after the 2nd dose. Plasma was tested for IgG and IgA to full-length spike and its receptor-binding domain (RBD) and for anti-spike IgA1 and IgA2. The titers of antigen-specific Ig are expressed in IU/ml, except for IgA2 that are expressed in AU (see Materials and Methods). LoD is indicated by a dotted line: LoD (spike IgG) = 12, LoD (RBD IgG) = 13.8, LoD (spike IgA) = 54.22, LoD (RBD IgA) = 54.08, LoD (IgA1) = 74.64; LoD (IgA2) = 0.009. The box plots show the interquartile range, the horizontal lines show the median values, and the whiskers indicate the minimum-to-maximum range. Each dot corresponds to an individual subject. Log scale on y axis (except for IgA2). P-values were determined using the Friedman test with the Dunnett's multiple comparison test.

 © 2022 The Authors

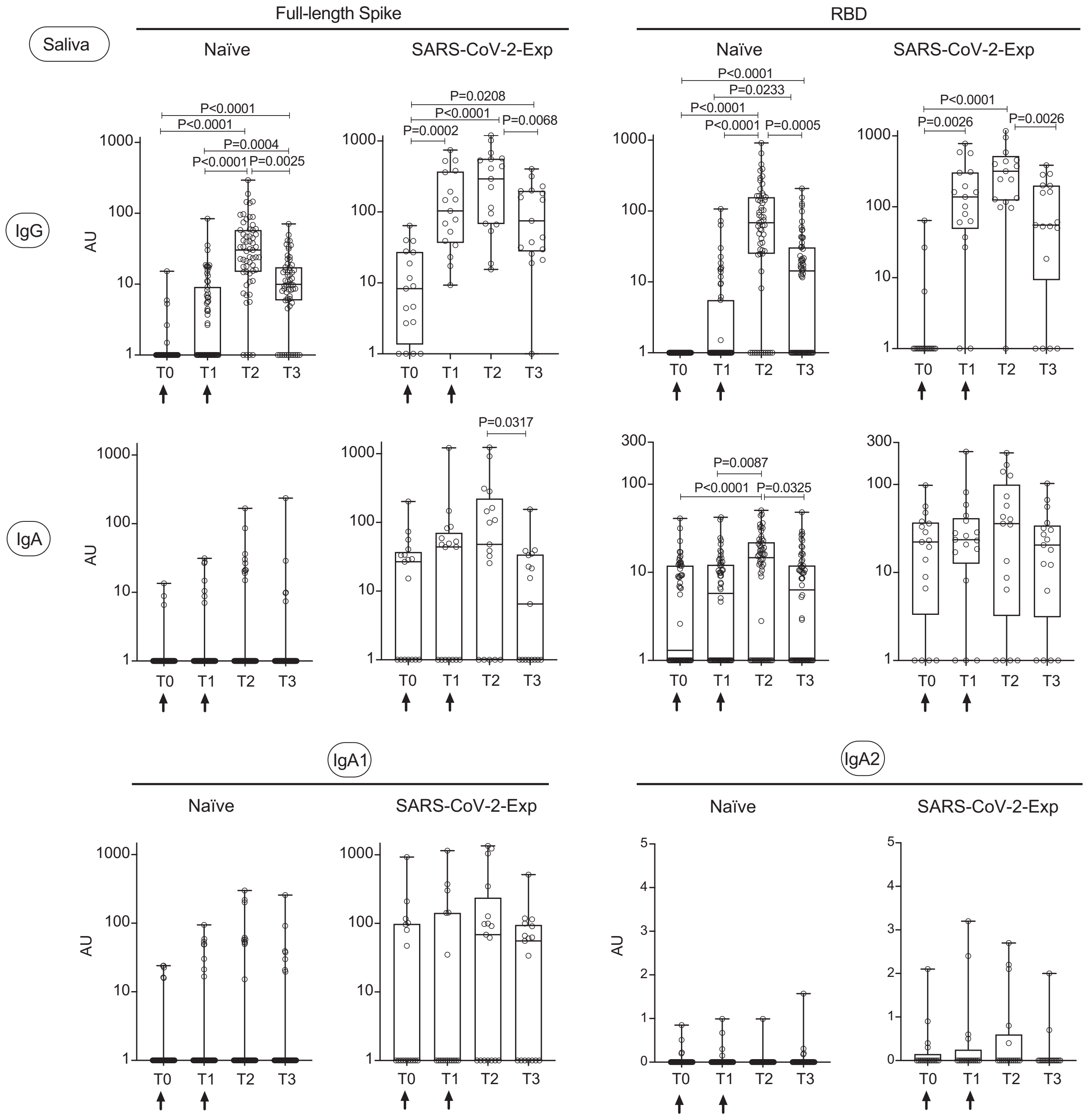

Figure EV4. SARS-CoV-2-specific IgG, IgA, and IgA1 levels decrease in saliva three months after BNT162b2 vaccination.

SARS-CoV-2-specific antibody responses were measured in the saliva of vaccinated naïve ($n = 59$) and SARS-CoV-2-Exp ($n = 17$) subjects at different time points: at the time of the first (T0) and the 2nd dose vaccine dose (T1) (indicated with an arrow), at 7–10 days (T2) and 3 months (T3) after the 2nd dose. Saliva was tested for IgG and IgA to full-length spike and its receptor-binding domain (RBD) and for anti-spike IgA1 and IgA2. The titers of antigen-specific Ig were normalized by dividing the values of SARS-CoV-2-specific Ig by total IgA or total IgG concentrations of each sample. The normalization was applied only to values higher than LoD. The adjusted values are expressed in AU. The box plots show the interquartile range, the horizontal lines show the median values, and the whiskers indicate the minimum-to-maximum range. Each dot corresponds to an individual subject. Log scale on y axis (except for IgA2). *P*-values were determined using the Friedman test with the Dunnett's multiple comparison test.

© 2022 The Authors
